# Risk of being granted disability pension among incident cancer patients before and after a structural pension reform: A Danish population-based, matched cohort study

**DOI:** 10.5271/sjweh.3883

**Published:** 2020-07-01

**Authors:** Pernille Pedersen, Maria Aagesen, Lars Hermann Tang, Niels Henrik Bruun, Ann-Dorthe Zwisler, Christina M Stapelfeldt

**Affiliations:** Section for Clinical Social Medicine and Rehabilitation, Department of Public Health, Aarhus University, Aarhus C, Denmark; DEFACTUM, Social & Health Services and Labor Market, Central Region Denmark, Aarhus C, Denmark; Knowledge Centre for Rehabilitation and Palliative Care, University of Southern Denmark and Odense University Hospital, Denmark; Departmennt of Rehabilitation and Nutrition, Faculty of Health and Technology, Metropolitan University College, Copenhagen, Denmark; Unit of Clinical Biostatistics, Aalborg University Hospital, Aalborg, Denmark

**Keywords:** Key terms Denmark, Disability Pension Act, quality of life, return to work, vocational rehabilitation

## Abstract

**Objective:**

This study aimed to examine the risk of being granted a disability pension (DP) among incident cancer patients up to five years after diagnosis compared to a match control group, before and after the structural reform of the Danish Disability Pension Act in 2013.

**Methods:**

All 20–60-year-old incident cancer-diagnosed individuals from 2000 to 2015 were identified in the Danish Cancer Registry. A control group, not previously diagnosed with cancer, was identified in Statistics Denmark matched by gender, age, education, and household income. Risk differences (RD) in cumulative incidence proportions of being granted a DP between cancer patients and controls were analyzed before and after the reform.

**Results:**

In total, 111 773 incident cancer patients and 506 904 controls were included in the study. Before reform 10 561 cancer patients and 11 231 controls were granted DP; and 2570 cancer patients and 2646 controls were granted DP after the reform. The adjusted RD of being granted DP was significantly higher for cancer patients versus controls at all time points before the reform. The RD increased the most during the first (RD 3.6, 95% CI 3.5–3.7) and second (RD 7.2, 95% CI 7.0–7.4) follow-up year and levelled off the remaining three years. After the reform, the adjusted RD were lower for all 1–5 follow-up years compared to before the reform (RD range 2.8–7.7, 95% CI 2.6–8.1).

**Conclusion:**

The 2013 reform of the Disability Pension Act reduced the risk of cancer patients being granted DP. The impact on a personal level should be further explored.

In the Nordic countries, 37% of all patients diagnosed with cancer are of working age, ie, 2–64 years (1). The possibility to engage in paid work is in general an important contributor to quality of life also for the increased prevalence of working-age cancer survivors as it restores identity and feelings of normality and solves financial concerns (2, 3). Hence, the motivation to return to work is high (4) and reflected in an average of 60% (range 24–94%) of all cancer survivors actually returning to work (5). However, the lower range of return-to-work (RTW) successes implies that cancer survivors may face complications and disabilities that call for vocational rehabilitation (6).

Between 1980 and 2001, the risk of early retirement pension was 55–60% higher among Danish cancer patients compared to a matched control group (7). Since then, the focus on vocational rehabilitation for this group has grown both nationally (8, 9) and internationally (10).

In the framework presented by Labriola (11), legislation is illustrated as a structural factor that overall tries to support individuals in their attempt to recover and return to work. In the Nordic welfare model, a generous benefit system offers financial security to sickness absentees who are unable to work. In cases of permanent work disability that inhibits work attendance, pensions are available. However, life courses have changed in the last century, moving toward a shorter work career due to more time spent on education before entering the workforce and an earlier retirement age. Compounded by the demographic development towards aging populations, countries can no longer afford their current social benefit schemes and must introduce reforms that increase retirement age and facilitate an inclusive labor market allowing people with <100% work ability to participate (12). It is expected that the prevalence of chronic diseases will increase within an aging and inclusive workforce, which may in turn increase sick leave levels and lower productivity (13). In Austria, the retirement age was postponed by approximately two and three years for men and women, respectively (14), which in fact increased employment. However, spillover effects were seen especially in an increase in unemployment benefits, although disability insurance claims were largely unaffected. In Sweden, a social insurance reform was introduced in 2008 that decreased entitlement to sickness benefits and disability pension (DP) (15). Overall, a reduced number of individuals were granted sickness benefits and DP in 2011 versus 2004, but more went on statutory and employment pensions. Another Swedish study investigated the 1995–2010 sick leave rate among employees aged ≥65 years (16). Even though the prevalence of ≥65-year old employees increased within this timeframe, sick leave rates were lower in 2010 than in 1995.

In January 2013, a reform of the Danish Disability Pension Act was introduced that aimed to reduce the number of granted DP, in particular among persons ≤40 years (17). The background for introducing this reform was an increasing incidence of granted DP especially to young individuals due to mental causes. The ideology behind the reform was to send a clear signal to young adults that they are not being abandoned and forgotten but supported and offered rehabilitation to improve their quality of life and ability to contribute to society. The reform also introduced multidisciplinary rehabilitation teams within each municipality to initiate rehabilitation efforts for these young adults at risk of being marginalized. The impact of this reform for cancer patients has not previously been studied, and to our knowledge no other studies on DP reforms targeted at primarily young adults have been studied.

As presented in the framework by Labriola (11), several factors related to personality trait, health, and the work environment have been identified as risk factors for DP. In the Danish register-based study by Carlsen et al (7), granted DP were more frequently seen among incident lymphomas and prostate and ovary cancers. Moreover, old age (18), unemployment, and long-term sick leave also influence work termination (19, 20). Incident cancer patients are in general more comorbid than the background population (21), which may affect their work ability and thus increase the risk of premature exit from the labor market (22). Ethnic minority groups have a higher cancer incidence and poorer survival rates than the majority group in western developed countries (23). Moreover, work conditions for low income and low-level educational jobs have been hypothesized as an explanation for the social inequality in cancer survivors’ permanent withdrawal from the labor market (24).

From the perspective of the dynamics of work disability prevention, it is therefore important to study, when taking other risk factors into account, how a structural reform affected incident cancer patients’ labor market prospects. Thus, this study examined the risk of being granted a DP among incident cancer patients up to five years after diagnosis compared with a matched control group, before and after a 2013 structural reform of the Danish Disability Pension Act.

## Methods

### Study population

All incident first-time cancer-diagnosed individuals aged 20–60 years in the period January 2000 to December 2015 were identified in the Danish Cancer Registry (CAR) (25) along with date of diagnosis. Since 1943, all incident cancers have been registered in CAR. Only diagnoses categorized according to Nordic cancer statistics (NORDCAN) were included. Thus, non-melanoma skin cancer was excluded as documentation of this condition is considered heterogeneous and incomplete (26).

A control group was identified in Statistics Denmark in 1:5 ratio matched on gender, age (ten-year age strata), highest completed education (primary/high school, vocational education, education <3 years, bachelor degree, and master degree), and household income (≤ -60 395, -60 394− -20 132, -20 131– -1, 0–20 131, 20 132–40 263, 40 264–60 394, ≥60 395 euros), defined at the time of diagnosis for the cancer patient. The matched controls had not previously been diagnosed with cancer except for non-melanoma skin cancer.

To ensure that the controls were indeed cancer free, CAR provided the personal identity number to Statistics Denmark of all Danes who, prior to 2000, had a cancer diagnosis. Thus being registered in CAR prior to 2000 prohibited an individual from being eligible as a control.

Matched controls were assigned the same baseline date as the diagnosis date of the cancer patient.

### Data sources and procedures

For both the cancer patients and controls, a unique personal identification number assigned to every Danish resident enabled linkage of information from multiple registers: CAR (25), Statistics Denmark, the Danish National Patient Register (DNPR) (27), and the Danish Register for Evaluation of Marginalization (DREAM) (28).

### Outcome measures

The cumulative incidence proportion (CIP) of granted DP was measured from baseline and up to a maximum of five years’ follow-up. Information about DP was identified in DREAM, which records all social transfer payments on a weekly basis and encompasses information from July 1991 to the present (29). The granting of DP is registered in that particular week where the beneficiary receives the pension and will continue to do so until old age pension, emigration or death occurs. Thus three types of events were treated as competing risks in the analyses.

Data from DREAM has previously been validated against workplace-registered data on sick leave (28, 30) and self-reported information on type of income (28), and both studies found that DREAM provides valid data.

### Potential confounders

Ethnicity was identified in DREAM and categorized as Danish, Western (except Danish), and non-Western.

Comorbidity was based on the Charlson Comorbidity Index (CCI) for a period of five years prior to baseline (31–33). The DNPR provided data on 19 selected somatic comorbidities, scored on a 3-point severity scale to create the CCI, which was then categorized as 0, 1–2 and ≥3.

Long-term sickness absence (≥4 weeks) 12–24 months prior to baseline was identified in DREAM and based on weeks with sickness benefits and reported in three categories (0, 1–26 and 27–52 weeks). A 12-month wash out period presiding cancer diagnosis was chosen due to potential confounding from reduced workability one year before the cancer diagnosis (34).

### Statistical analyses

The frequencies of matching variables and potential confounders were reported for the cancer and control populations. For descriptive purposes only, the cancer diagnoses were reported and categorized according to NORDCAN [breast, upper-gastro-and-intestines (UPGI), melanoma skin, colorectal, male genitals, lung, gynecological, brain and central nervous system (CNS), blood, kidney and bladder, other] (26).

The CIP of granted DP was counted between baseline and up to 1–5-year follow-up for the cancer and the control groups. Differences in CIP between the cancer and control groups were interpreted as risk differences (RD) and pseudo observations in generalized linear regressions were used for estimation (35, 36).

Entry was defined as the date of cancer diagnosis for the incident cancer patients and their matched controls accordingly. The end of follow-up was defined by the week where DP was granted, the occurrence of competing risks (old age pension, emigration or death) or end of 1–5-year follow-up, which ever occurred first.

During follow-up in the present study, the Danish Disability Pension Act was reformed in January 2013, by which individuals aged ≤40 years in principle no longer could be granted a DP. To account for the reform, delayed entry was applied for cancer patients diagnosed before January 2013 and still at risk of being granted DP by January 2013 and onwards. Adjusted for matching variables, crude and adjusted RD were presented.

To assess whether RD significantly changed following the reform, RD were subtracted (after minus before 2013) and 95% confidence intervals (CI) were calculated by the corresponding RD standard errors.

Moreover, 5-year CIP for the cancer population and RD stratified on the matching variables were estimated.

The significance level was set at P<0.05. STATA version 15.1 (StataCorp, College Station, Texas, USA) was used as statistical software.

### Ethics

The Danish Data Protection Agency approved this study (1-16-02-445-16). According to Danish law, approval from the Danish National Committee on Biomedical Research Ethics was not needed as this is only provided for projects using biological material or involving biomedical treatment. All procedures performed in studies involving human participants were in accordance with the ethical standards of the institutional and/or national research committee and with the 1964 Helsinki Declaration and its later amendments or comparable ethical standards (37).

## Results

**Figure 1:**
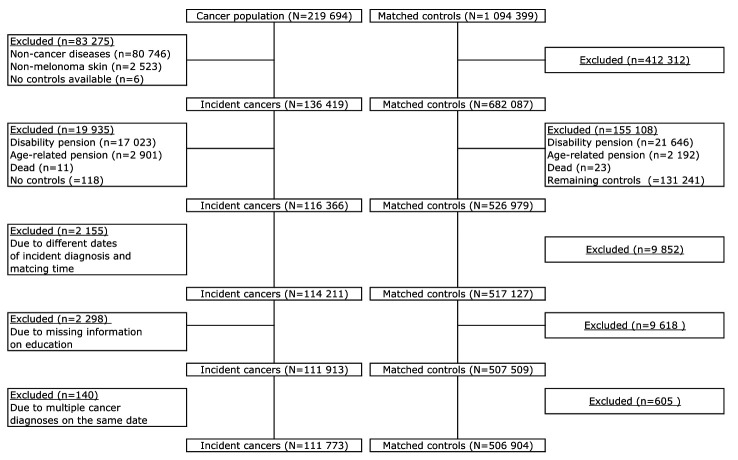
Flow chart of the exclusion process from initial to final study sample.

A total of 219 694 20–60-year-old individuals were identified in CAR between 2000 and 2015, and 1 094 399 controls were identified in the matching procedure. Of those, 83 275 (38%) cancer patients were excluded; primarily due to non-cancer diseases (ie, precancerous lesions) and non-melanoma skin cancer diagnosis. That resulted in an exclusion of 412 312 (38%) matched controls. In total, 111 773 incident cancer patients and 506 904 matched controls met the inclusion criteria and were included in the study and followed for up to five years (figure 1).

More 50–60-year-olds were present in the cancer (58.0%) than control (55.8%) population. Also, more cancer patients than controls had primary or high school as their highest achieved education and earned €0–20 131 per year (table 1). More ethnic Danes were diagnosed with cancer than western and non-western residents. Cancer patients had more comorbidity the presiding five years before cancer diagnosis and spend more time on sick leave during the year prior to the wash-out period than the controls. Breast and UPGI cancer were the most incident cancer types, whereas kidney and bladder as well as other cancers had the lowest incidence (table 1).

**Table 1:** Matching variables and baseline characteristics of the study population.

	Cancer N=111 773	Controls N=506 904	Total N=618 677
		
	N (%)	N (%)	N (%)
Age-matching group			
20–29	5658 (5.1)	27 938 (5.5)	33 596 (5.4)
30–39	13 124 (11.7)	63 835 (12.6)	76 959 (12.4)
40–49	28 193 (25.2)	132 440 (26.1)	160 633 (26.0)
50–60	64 798 (58.0)	282 691 (55.8)	347 489 (56.2)
Gender-matching group			
Female	62 405 (55.8)	281 720 (55.6)	344 125 (55.6)
Male	49 368 (44.2)	225 184 (44.4)	274 552 (44.4)
Education-matching group			
Primary & high school	32 234 (28.8)	132 123 (26.1)	164 357 (26.6)
Vocational education	45 218 (40.5)	209 344 (41.3)	254 562 (41.2)
Short further education	5295 (4.7)	25 355 (5.0)	30 650 (5.0)
Bachelor education	20 770 (18.6)	99 661 (19.7)	120 431 (19.5)
Long further education & research	8295 (7.4)	40 421 (8.0)	48 677 (7.9)
Income-matching group (euros)			
≤-60 395	70 (0.1)	339 (0.1)	409 (0.1)
-60 394– -20 132	176 (0.2)	861 (0.2)	1037 (0.2)
-20 131– -1	1517 (1.4)	7426 (1.5)	8943 (1.5)
0–20 131	28 672 (25.7)	113 460 (22.4)	142 132 (23.0)
20 132–40 263	64 034 (57.3)	299 177 (59.0)	363 211 (58.7)
40 264–60 394	13 198 (11.8)	65 237 (12.9)	78 435 (12.7)
≥60 395	4106 (3.7)	20 404 (4.0)	24 510 (4.0)
Ethnicity			
Danish	103 841 (92.9)	461 910 (91.1)	565 751 (91.5)
Western	3020 (2.7)	15 217 (3.0)	18 237 (3.0)
Non-western	3660 (3.3)	24 389 (4.8)	28 049 (4.5)
Unknown	1252 (1.1)	5388 (1.1)	6640 (1.1)
Comorbidity 5 years before			
0	103 006 (92.2)	481 912 (95.1)	584 918 (94.5)
1–2	7771 (7.0)	22 808 (4.5)	30 579 (4.9)
≥3	996 (0.9)	2184 (0.4)	3180 (0.5)
Sick leave 2 years before index date (weeks)			
0	93 930 (84.0)	433 626 (85.5)	527 556 (85.3)
1–26	15 419 (13.8)	64 121 (12.7)	79 540 (12.9)
27–52	2424 (2.2)	9157 (1.8)	11 581 (1.9)
NORDCAN cancer site			
Breast	23 550 (21.1)		
Upper-gastro, intestines	11 968 (10.7)		
Melanoma skin	11 913 (10.7)		
Colorectal	9958 (8.9)		
Male genitals	9905 (8.9)		
Lung	8359 (7.5)		
Gynaecological	8219 (7.4)		
Central nervous system	8167 (7.3)		
Blood	8069 (7.2)		
Kidney & bladder	6403 (5.7)		
Other	5262 (4.7)		

During the five years follow-up before the reform a total of 10 561 cancer patients were granted DP and 41 718 controls. The adjusted RD of being granted a DP for cancer patients was significantly higher than the controls at all time points (table 2). The RD of granted pensions between the controls and cancer patients were most pronounced within the first and second follow-up year (RD 3.60, 95% CI 3.46–3.74 and RD 7.20, 95% CI 7.01–7.40, respectively) and levelled off the remaining three years.

**Table 2:** Risk differences (RD) in cumulated incidence proportions (CIP) of being granted disability pensions before and after January 2013 (time of structural reform). [CI=confidence interval.]

	Cancer		Non-cancer		Cancer	RD		RD difference after-before (95% CI) ^c^
			
	Disability pensions	Competing risk ^a^	Disability pensions	Competing risk ^a^	CIP % (95% CI)	% (95% CI) ^b^	% (95% CI) ^c^	
Before reform								
Year 0–1	3829	6364	2704	11 769	4.47 (4.33–4.61)	3.63 (3.49−3.77)	3.60 (3.46−3.74)	
Year 0–2	7349	14 356	5261	17 260	8.87 (8.67–9.06)	7.21 (7.02–7.41)	7.20 (7.01–7.40)	
Year 0–3	8944	23 522	7628	20 374	11.03 (10.81–11.24)	8.58 (8.36–8.79)	8.57 (8.35–8.79)	
Year 0–4	9933	32 486	9573	22 678	12.54 (12.31–12.78)	9.37 (9.14–9.6)	9.37 (9.14–9.61)	
Year 0–5	10 561	41 718	11 231	24 928	13.55 (13.31–13.79)	9.71 (9.46–9.96)	9.73 (9.48–9.97)	
After reform								
Year 0–1	696	993	368	2172	3.09 (2.85–3.33)	2.80 (2.56–3.05)	2.82 (2.57–3.06)	-0.78 (-1.06– -0.50)
Year 0–2	1496	3076	875	3906	5.90 (5.58–6.21)	5.29 (4.97–5.61)	5.33 (5.01–5.65)	-1.87 (-2.24– -1.50)
Year 0–3	2001	6101	1440	5148	7.48 (7.14–7.82)	6.55 (6.20–6.89)	6.59 (6.24–6.94)	-1.98 (-2.39– -1.57)
Year 0–4	2331	9367	2027	6142	8.54 (8.17–8.90)	7.25 (6.88–7.62)	7.29 (6.92–7.66)	-2.08 (-2.52– -1.64)
Year 0–5	2570	14 366	2646	7583	9.28 (8.91–9.66)	7.63 (7.25–8.01)	7.68 (7.29–8.06)	-2.05 (-2.51– -1.59)

a Death, age-related pension and emigration.

b Adjusted: matching variables (gender, age, education and income).

c Adjusted: matching variables plus (ethnicity, comorbidity and sick leave).

After the reform, 2570 and 2646 cancer patients and controls were granted DP, respectively. The CIP of being granted a disability pension for cancer patients were lower for all follow-up years than before the reform, ranging from 3.09% (95% CI 2.85–3.33) within the first year to 9.28% (95% CI 8.91–9.66) within the five years follow-up (table 2). The adjusted RDs were statistically significantly smaller after 2013 than before, ranging from RD difference -0.78 (95% CI -1.02– -0.50) to RD difference -2.05 (95% CI -2.51– -1.59) (table 2).

Within the first three follow-up years, the stratified analyses showed a tendency of higher RD between control and cancer men than women granted DP. Within the remaining two follow-up years, this difference levelled off (table 3). After the reform, the difference between men and women remained throughout the five years but at a lower level than before 2013. The younger the age, the smaller the RD observed – both before and after the reform. For those <40 years, the RD were approximately halved after the reform compared to before. For the age groups >39 years, a reduction in RD was seen after the reform but less pronounced than for those <40 years. For the 50–60-year-old group, the RD remained the same after the reform from the second until the fifth follow-up year up (table 3). The reform seemed to reduce the difference in RD for those with the highest achieved educational level and annual income. Whereas for the low-educated and small-income groups, the reduction in RD after the reform were much smaller than for the more socioeconomically fortunate. For those with a negative yearly income, the tendencies departed somewhat for the low socioeconomical groups and were more comparable with the well-educated and high-income groups.

**Table 3:** Cumulated incidence proportions (CIP) from diagnosis, up until 5 years after, and risk differences (RD) in CIP between non-cancer and cancer groups before and after 2013 reform, stratified on matching variables.

	Before January 2013 reform		After January 2013 reform	
	
	Cancer CIP	Adjusted for matching variables	Cancer CIP	Adjusted for matching variables
			
	% (95% CI)	RD % (95% CI)	% (95% CI)	RD % (95% CI)
Gender				
Female				
Year 0–1		2.75 (2.58–2.91		2.04 (1.76–2.32)
Year 0–2		6.35 (6.10–6.60)		4.13 (3.76–4.50)
Year 0–3		8.08 (7.79–8.37)		5.42 (5.00–5.83)
Year 0–4		9.16 (8.84–9.47)		6.30 (5.85–6.74)
Year 0–5	13.56 (13.23–13.89)	9.65 (9.32–9.98)	8.36 (7.90 -8.82)	6.77 (6.31–7.24)
Male				
Year 0–1		4.75 (4.52–4.98)		3.86 (3.42–4.30)
Year 0–2		8.31 (8.00–8.61)		6.89 (6.34–7.45)
Year 0–3		9.21 (8.87–9.54)		8.11 (7.51–8.71)
Year 0–4		9.64 (9.28–9.99)		8.57 (7.95–9.19)
Year 0–5	13.54 (13.18–13.90)	9.78 (9.42–10.15)	10.58 (9.95–11.22)	8.81 (8.18–9.45)
Age (years)				
20–29				
Year 0–1		1.50 (1.11–1.90)		0.61 (0.18–1.04)
Year 0–2		2.92 (2.37–3.48)		1.52 (0.90–2.14)
Year 0–3		3.84 (3.19–4.49)		2.36 (1.62–3.10)
Year 0–4		4.54 (3.82–5.26)		2.60 (1.83–3.38)
Year 0–5	6.17 (5.40–6.94)	5.08 (4.30–5.86)	3.37 (2.58–4.17)	2.79 (1.99–3.59)
30–39				
Year 0–1		1.40 (1.15–1.67)		0.77 (0.45–1.09)
Year 0–2		3.45 (3.04–3.85)		1.53 (1.11–1.96)
Year 0–3		4.89 (4.41–5.38)		2.23 (1.73–2.73)
Year 0–4		5.71 (5.18–6.25)		2.66 (2.12–3.19)
Year 0–5	8.62 (8.03 -9.20)	6.66 (6.07–7.25)	3.91 (3.34–4.48)	3.00 (2.43–3.58)
40–49				
Year 0–1		3.10 (2.84–3.35)		1.75 (1.40–2.11)
Year 0–2		6.36 (5.99–6.72)		3.51 (3.04–3.98)
Year 0–3		7.94 (7.52–8.36)		4.37 (3.86–4.89)
Year 0–4		9.06 (8.61–9.52)		5.02 (4.47–5.57)
Year 0–5	13.26 (12.78–13.74)	9.57 (9.08–10.06)	6.85 (6.28–7.42)	5.46 (4.89–6.04)
50–60				
Year 0–1		4.52 (4.31–4.72)		4.49 (4.04–4.94)
Year 0–2		8.75 (8.47–9.03)		8.26 (7.69–8.84)
Year 0–3		10.04 (9.73–10.35)		10.02 (9.39–10.65)
Year 0–4		10.69 (10.36–11.02)		10.92 (10.26–11.58)
Year 0–5	15.32 (14.98–15.66)	10.81 (10.47 -11.16)	13.57 (12.90–14.25)	11.30 (10.62–11.98)
Education				
Primary/high school				
Year 0–1		5.39 (5.08–5.70)		4.99 (4.36–5.62)
Year 0–2		9.76 (9.34–10.19)		8.16 (7.38–8.93)
Year 0–3		11.50 (11.03–11.97)		9.76 (8.92–10.60)
Year 0–4		12.26 (11.76–12.76)		10.55 (9.67–11.43)
Year 0–5	18.64 (18.12–19.15)	12.52 (11.99–13.04)	13.95 (13.05–14.84)	11.02 (10.11–11.93)
Vocational education				
Year 0–1		3.73 (3.51–3.95)		2.94 (2.55–3.34)
Year 0–2		7.51 (7.20–7.82)		6.03 (5.49–6.56)
Year 0–3		8.91 (8.57–9.25)		7.38 (6.80–7.96)
Year 0–4		9.70 (9.34–10.07)		8.16 (7.55–8.78)
Year 0–5	13.47 (13.09–13.85)	10.06 (9.67–11.44)	10.15 (9.52–10.78)	8.59 (7.95–9.22)
Education <3 years				
Year 0–1		1.97 (1.48–2.46)		1.69 (0.87–2.50)
Year 0–2		4.38 (3.65–5.11)		3.42 (2.33–4.51)
Year 0–3		5.15 (4.33–5.96)		4.33 (3.13–5.54)
Year 0–4		5.94 (5.04–6.84)		4.66 (3.39–5.92)
Year 0–5	8.64 (7.71–9.57)	6.16 (5.21–7.11)	5.78 (4.48–7.08)	4.91 (3.60–6.22)
Bachelor degree				
Year 0–1		2.01 (1.76–2.26)		1.00 (0.66–1.34)
Year 0–2		4.86 (4.48–5.24)		2.57 (2.08–3.07)
Year 0–3		5.98 (5.55–6.41)		3.63 (3.05–4.20)
Year 0–4		6.90 (6.43–7.37)		4.37 (3.75–4.99)
Year 0–5	9.54 (9.05–10.03)	7.34 (6.84–7.83)	5.71 (5.07–6.36)	4.68 (4.03–5.34)
Master degree				
Year 0–1		1.19 (0.88–1.51)		0.69 (0.27–1.11)
Year 0–2		3.06 (2. 57–3.55)		1.20 (0.66–1.73)
Year 0–3		3.73 (3.17–4.28)		1.72 (1.10–2.33)
Year 0–4		4.34 (3.72–4.95)		1.91 (1.26–2.56)
Year 0–5	6.25 (5.60–6.90)	4.75 (4.09–5.40)	2.36 (1.67–3.04)	2.10 (1.41–2.79)
Income (euros per year)				
≤-60 395				
Year 0–1		3.32 (-1.23–7.87)		-0.03 (-0.32–0.27)
Year 0–2		6.79 (0.33–13.26)		-0.13 (-0.64–0.39)
Year 0–3		6.34 (-0.16–12.83)		-0.16 (-0.83–0.50)
Year 0–4		6.27 (-0.21–12.76)		4.71 (-4.82–14.25)
Year 0–5	9.50 (1.78–17.22)	8.50 (0.79–16.21)	4.79 (4.71–14.29)	4.72 (-4.80–14.25)
-60 394– -20 130				
Year 0–1		2.27 (-0.37–4.91)		-0.21 (-46–0.03)
Year 0–2		5.88 (1.60–10.16)		-2.26 (-2.91–7.43)
Year 0–3		6.02 (1.47–10.57)		1.76 (-3.45–6.98)
Year 0–4		6.61 (1.76–11.46)		1.60 (-3.61–6.81)
Year 0–5	9.74 (4.72–14.76)	7.63 (2.48–12.78)	4.99 (-2.26–12.24)	2.63 (-4.77–10.04)
-20 131– -1				
Year 0–1		3.83 (2.70–4.96)		2.32 (-0.11–4.74)
Year 0–2		5. 11 (3.67–6.55)		6.05 (2.49–9.61)
Year 0–3		5.07 (3.49–6.65)		6.13 (2.50–9.77)
Year 0–4		4.82 (3.12–6.51)		5.74 (2.11–9.37)
Year 0–5	8.98 (7.36–10.59)	3.52 (1.78–5.25)	8.52 (4.73–12.30)	6.20 (2.40–10.00)
0–20 131				
Year 0–1		7.02 (6.65–7.38)		6.73 (5.86–7.60)
Year 0–2		11.74 (11.26–12.22)		10.26 (9.21–11.30)
Year 0–3		13.47 (12.94–14.01)		11.91 (10.79–13.03)
Year 0–4		14.35 (13.78–14.92)		12.64 (11.48–13.80)
Year 0–5	22.81 (22.24–23.38)	14.55 (13.96–15.15)	18.01 (16.83–19.19)	13.22 (12.03–14.42)
20 132–40 263				
Year 0–1		2.60 (2.45–2.75)		2.28 (1.99–2.56)
Year 0–2		6.13 (5.90–6.36)		4.77 (4.39–5.16)
Year 0–3		7.58 (7.32–7.85)		6.11 (5.69–6.54)
Year 0–4		8.48 (8.20–8.76)		6.94 (6.49–7.39)
Year 0–5	11.02 (10.73–11.31)	8.96 (8.66–9.26)	8.74 (8.28–9.21)	7.31 (6.84–7.78)
40 264–60 394				
Year 0–1		1.12 (0.86–1.37)		0.99 (0.61–1.37)
Year 0–2		2.71 (2.31–3.10)		2.72 (2.15–3.29)
Year 0–3		2.98 (2.55–3.41)		3.48 (2.85–4.11)
Year 0–4		3.21 (2.75–3.66)		3.89 (3.22 -4.57)
Year 0–5	4.96 (4.49–5.43)	3.36 (2.89–3.84)	3.68 (2.98–4.37)	4.19 (3.49–4.89)
≥60 395				
Year 0–1		0.76 (0.37–1.15)		0.19 (-0.24–0.61)
Year 0–2		2. 39 (1.72–3.05)		0.79 (0.14–1.43)
Year 0–3		2.89 (2.14–3.63)		1.31 (0.53–2.09)
Year 0–4		3.28 (2.48–4.09)		1.42 (0.61–2.24)
Year 0–5	4.76 (3.93–5.59)	3.46 (2.61–4.30)	0.81 (-0.02–1.63)	1.45 (0.61–2.29)

Focusing solely on the cancer population, the CIP of being granted a DP within the five-year follow-up period were reduced after the reform for all matching variables compared with the CIP before the reform. Unlike the reductions in RD after the reform, the reductions in CIP were not particularly associated with the different levels of the various matching variables. The smallest reduction in CIP (8.98–8.52 = 0.46% points) was observed for the -€20 131– -1 income-strata and the highest was observed for the 40–49-year age-strata (13.26–6.85 = 6.41% points) (table 3).

## Discussion

This Danish population-based study compared the CIP of being granted a DP among incident cancer patients up to five years after diagnosis, before and after a structural reform of the Danish Disability Pension Act in January 2013. Overall, we found the RD between controls and cancer patients of being granted a DP were reduced after 2013. This was especially the case for socio­economically fortunate groups. For the cancer group, the CIPs were overall reduced after the reform and these reductions were less dependent of socioeconomic variables than among the controls.

Labriola’s Dynamic Work Disability Model (11) proposed different factors that may affect pathways leading to RTW or termination of work, representing the two possible extremes in the model. Structural factors such as legislation are illustrated as a RTW-promoting mechanism (11). The overall purpose of the 2013 structural reform of the Disability Pension Act was to reduce the number of granted DP in particular among 18–40-year-olds (17). As our findings showed an overall reduced risk of being granted DP among cancer patients after (versus before) 2013, municipal social workers responsible for the granting of DP seem to have translated the legislation according to the intension of the reform, ie, in favor of vocational rehabilitation. However, as the risks were reduced irrespective of gender, age, education and income among the cancer patients within the five-year follow-up, it may point toward a generally decreased disability among cancer survivors and thereby a reduced need for permanent withdrawal from the labor market. However, improvements in early diagnosis and treatments are mentioned as factors responsible for reduced mortality rates (38), whereas the increased prevalence of cancer survivors may experience increased levels of comorbidity, physical, and mental late effects that may reduce work ability (39). Looking at the reductions in RD from before to after 2013, the results pointed in different directions. On one hand, cancer patients were still more likely to be granted DP than controls but, on the other hand, this had also to do with increased risks of DP among the controls depending on their socioeconomic status. The introduction of municipal rehabilitation teams has put emphasis on vocational rehabilitation in general; social workers may be more aware of offering other public benefits than DP targeted to sustain work ability and employability. This is in line with experiences gained from the introduction of social reforms in other countries, where spill-over effects were observed such as increased unemployment rates when retirement age was postponed (14) and an increase of statutory pensions were seen when the entitlement to DP and sickness benefits was reduced (15). It may be an indication of a fulfillment of the reform’s overall purpose, namely to increase attachment to the labor market, and thus supporting the assumptions put forward in Labriola’s conceptual framework (11). It is, however, important to further investigate if the reform merely maintains individuals on social benefits, other than DP, with no work prospects. From 2013 until the present, an increased CIP of granted DP has been observed in Denmark. This may be a delayed effect of initiated rehabilitation efforts that ultimately did not reveal any work ability (38–40).

Along with the reform, regulations of the employment scheme for partly work-disabled (flexi-job) were also carried out. Special vocational rehabilitation teams within the municipal social services were formed to ensure early interdisciplinary input and support the work-disabled to return to work in ordinary or modified jobs, thus avoiding DP. However, a reduction in granted pensions does not inform about how the work-disabled in general, and cancer patients in particular, experience these new initiatives and whether work participation has indeed increased. Therefore knowledge is needed about those work-disabled who, before 2013, would have been granted a DP but currently are offered alternative means of economic and rehabilitative support. Studies should look into this potentially vulnerable group of individuals. A cancer diagnosis often leads to new perspectives on life. For some, a re-evaluation of work life (41) may lead to a desire to change occupation or engage in activities such as volunteer work and thus, in some cases, renounce the right to social benefits (including DP). The patient-centered perspective should therefore be further explored as to whether the reform gives individuals the opportunity to engage in activities that are perceived as meaningful and improving quality of life.

In the present study, the cancer patients were more likely to receive DP than controls in both time periods. Similar findings were identified in Carlsen et al’s Danish register-based study from 2008 (7), in which an increased risk of 60% and 55% in female and male cancer patients, respectively, was reported compared with matched controls. In Carlsen et al’s and the present study, adjustments for known risk factors related to the workplace environment were not possible due to the register-based design. The workplace arena is considered an important stakeholder in vocational rehabilitation (42). Within Labriola’s Dynamic Work Disability Model (11), management quality was identified as a possible modifying factor between person-related factors and the pathways between RTW and work termination. This was further substantiated in Feuerstein et al’s review (43) where work environment factors were also found to be important in RTW studies among cancer survivors. Along with the introduction of social reforms, it is clear that the involvement of the workplace arena is a requisite for RTW (13) as the work environment needs adjustments to sustain work ability among an aging and not 100% fit workforce, in particular in mentally and physically demanding professions. The workplace arena and its effect on cancer patients’ risk of DP should be further explored.

Carlsen et al (7) found that the risk of being granted DP increased with 9% and 8% per ageing year among women and men, respectively, compared with controls. We also found that the risks of DP increased with age among the cancer patients. Fortunately, few were diagnosed with cancer before the age of 40 (16.8%), and fewer were granted a DP in these age groups than among those >40 years both before and after 2013 in the present study. Despite these encouraging results, previous studies have reported that young adults with cancer often struggle during and after the RTW process to sustain work participation (44, 45). Young cancer patients typically experience problems with paying attention, forgetting and keeping up with work (45). Thus, even though young adults are able to return to work, they still need support afterwards to sustain their work participation. However, effective interventions to support sustained RTW and prevent permanent work disability in young cancer patients are scarce (45), and future research is warranted.

### Methodological considerations

This study has several strengths due to its population-based design and high-quality registry data from CAR (25), DNPR (27), and DREAM (9, 28), which limited the risk of selection and information bias. However, the design prohibited adjustments for workplace- and health-related factors such as pain, anxiety and depression, which may have led to confounded risk estimates. Furthermore, the DP reform in 2013 restricted the granting of DP to persons <40 years, which we would have liked to adjust for in the analyses. However, our matching procedure used 10-year age strata and thus, exact age at time of diagnosis and the granting of DP was unknown. We were therefore only allowed to perform delayed entry, ie, both the cancer and control groups had survived beyond January 2013 if diagnosed with cancer prior to that time point and had not yet been granted a DP. We suspect this may be the reason for the control group’s increased DP risk after January 2013, possibly leading to underestimated RD after that time point.

As social security schemes vary between countries, the transferability of our results may be limited to the Nordic region, which also has a tax-financed benefit system and is considered to offer generous systems compared with most other countries.

### Concluding remarks

This Danish population-based study showed that a 2013 structural reform of the Disability Pension Act reduced the risk for cancer patients to be granted a DP. The differences between controls’ and cancer patients’ risk of being granted a DP were significantly reduced after the reform. However, reductions were more pronounced among the socioeconomically fortunate versus unfortunate. The impact of the reduced number of granted DP on cancer patients’ quality of life is unknown. The patient-centered perspective should therefore be further explored as to whether the reform provides individuals the opportunity to engage in supportive activities and vocational rehabilitation that are perceived as meaningful and improving of quality of life.
